# The motivational effect of multicolored dental restoration on dental behavior of first preliminary school children

**DOI:** 10.1002/cre2.194

**Published:** 2019-06-19

**Authors:** Lojain Abdulaziz Melebari, Seba Essam Attas, Abla Arafa

**Affiliations:** ^1^ Dental Interns at Faculty of Dentistry Umm al‐Qura University Makkah Kingdom of Saudi Arabia; ^2^ Department of Pediatric Dentistry and Dental Public Health, Faculty of Oral and Dental Medicine Misr International University Cairo Egypt; ^3^ Faculty of Dentistry Umm al‐Qura University Makkah Kingdom of Saudi Arabia

**Keywords:** behavior management, multicolored restoration, plaque index

## Abstract

**Objectives:**

To assess the motivational effect of multi‐colored restoration on the anxiety level of pediatric patients at thedental clinic and its motivational effect on their oral hygiene status.

**Material and methods:**

A total of 30 participants.

**Results:**

Both groups revealed reduction in the anxiety level and improvement in their behavior at the dental office but did not reach significance. Plaque index showed a significant reduction per group and a near significance as compared between test groups.

**Conclusions:**

The use of the multicolored restoration could provide a potential advantage to improve the oral health status of children and might aid in enhancing their behavior at dental clinic particularly younger age groups.

**Bullet Points**

- Management of children behavior at the dental clinic always presents a serious challenge. The use of multi-colored restoration creates a joyful experience during the dental appointment that improves child's attitude toward dental procedure and enhances the child oral hygiene practice.
- The use of innovative multicolored restoration creates a joyful experience during the dental appointment that can enhance not only the oral hygiene practice of the child to preserve the cleanness of the inserted colored restoration but also improves his behavior and attitude toward dental procedure at the dental clinic.

## 1 INTRODUCTION

Managing the anxiety of young children via behavior control approaches presents critical provocation for pediatric dentists. Fear and anxiety have been recognized as a major public health dilemma in many countries and considered to be the main cause of behavior management problems and children avoidance of dental care; it might have also shown that the anxiety level increases in children having active caries (Gustafsson, Arnrup, Broberg, Bodin, & Berggren, 2007). It was reported that about 23.5% of children experience some sort of anxiety prior to dental procedure despite their age, gender, or the behavior management technique used with them. Increased anxiety level could yield reduced child cooperative behavior on the dental chair and possible affection of the final dental care quality (Pedrotti et al., 2015).

Accordingly, the health care professionals could find themselves obligated to convert their approaches to more complicated alternatives such as conscious sedation or even providing the dental intervention under general anesthesia (Patır Münevveroğlu, Ballı Akgöl, & Erol, 2014). Both techniques could yield unpleasant side effects as nausea, vomiting, sore throat, confusion, muscle aches, itching, and hypothermia with general anesthesia, whereas for conscious sedation, motor imbalance, gastro‐intestinal effects, and restlessness present possible complication (Rusch, Eberhart, Wallenborn, & Kranke, 2010).

The relationship between the dentist and his patients especially children represents a prime factor that should be as friendly as possible as to reduce the patient's fear and make him able to achieve a successful treatment procedure. The pediatric dentist could try multiple behavior management approaches to meet the child needs in order to alleviate the patient's anxiety and enhance child cooperation at the dental operatory (Vasiliki et al., 2016).

Permitting the child sense of control is an effective noninvasive behavior management technique that has been recognized to reduce disruptive behavior in the clinic. One of the nonverbal communication techniques invites child patient to share and enhance control via raising a hand to stop the dental procedure. In addition, giving the child the chance to choose the type or the color of the restoration that will be inserted in his teeth could be another successful approach (Sharma & Tyagi, 2011).

“Compomer” has been used as a primary teeth restoration since their appearance in the market in 1993. Multiple studies concluded that both hybrid composite and compomer have a similar success rate in children and a superior clinical success than glass ionomers (Arora, Arora, Srivastava, & Togoo, 2014; KrÄmer & Frankenberger, 2007).

An innovative photo‐polymerized multicolored compomer dental restorative material has been introduced into the pediatric dentistry field and revealed comparable bond strength characteristics with that of the conventional compomer (Güngör, Erdoğan, Yalçın‐Güngör, & Alkış, 2016). In contrast to the conventional compomer, a small amount of color and glitter particles was added to give different color shades as gold, silver, blue, pink, green, orange, and lemon provided that the filler content remained similar to that of the conventional compomer restorative material to avoid any alteration of the physical properties or the biocompatibility (Croll, Helpin, & Donly, 2004). All the inserted color pigments comply with food and cosmetics regulations and found to be toxicologically irrelevant giving good biological safety of multicolored restorative material. Furthermore, multicolored restoration still possesses a fluoride releasing ability and can be recharged by topical fluoride application, which makes it more suitable for placement in children particularly those with high caries risk (Arora et al., 2014).

Additionally, the assessment of the clinical performance of multicolored restoration showed that there was no significant difference found between the survival rate, marginal integrity, marginal discoloration, anatomic form, secondary caries development, and surface texture compared with regular compomer even after 12 months follow‐up period. Thus, the multicolored restorative material could be considered a good choice for placement in primary dentition (Ertugrul, Cogulu, Özdemir, & Ersin, 2010).

It has been suggested that the pediatric patients could become more cooperative and would have an enhanced positive attitude during the procedure when they consider the dental appointment a playful experience following their participation in the selection of the restoration color. Furthermore, the application of multicolored restoration could be accompanied with increased patient motivation toward their oral health care at home and lasting interest in their restored teeth (Arora et al., 2014). However, scanty researchers have studied the effect of multicolored restoration on reducing the anticipated children anxiety at the dental office via increasing their acceptance toward the dental procedure along with raising their awareness toward their oral hygiene. Therefore, this study aimed to assess the motivational effect of multicolored restoration on reducing dental anxiety level in pediatric patients at the dental clinic and on enhancing their adherence and effectiveness of oral hygiene behavior at home.

## 2 MATERIALS AND METHODS

### 2.1 Materials

The details of different restorative materials used in this study and participants grouping are shown in Table 1.

**Table 1:** Details of restorative materials used in the study and participants grouping

Restorative material				Manufacture
Group	Category	Product	Composition	
Group A	Multicolored compomer	Twinky Star®	Bis‐GMA, diurethane dimethacrylate, TEGDMA, carboxylic acid modified methacrylate, silicon dioxide, “BHT,” and camphoroquinone. Fillers: barium aluminum fluoro borosilicate glass. Dioxide particles and glimmer.	VOCO, dentalists, GmbH, Germany
Group B	Conventional compomer	Dyract ®Extra	Dimethacrylate, filler: ytterbium trifluoride, Al‐fluorosilicate glass, spheroid mixed oxide, initiators, stabilizers, and pigments.	Dentsply, USA

### 2.2 Ethical consideration

Ethical approval was obtained from the Institutional Review Board, Umm al‐Qura University, Faculty of Dentistry. Parents or legal guardian signed the consents former to their children participation in the study.

### 2.3 Sample size calculation

A power calculation indicated that minimum of 12 participants were needed in each group to demonstrate the effect at 80% power of significance and *p* < .05 using ClinCalc for sample size calculation.

### 2.4 Study design

Healthy children attending first preliminary school, with age range from 5‐ to 8‐year‐olds, and presented at the UQUDENT Teaching Hospital for their first dental visit were enrolled in the study during the period from September 2017 to February 2018. Children should have normal communication skills free from any mental, physical, or medical disability. The intraoral examination should reveal the presence of one or more simple carious cavity necessitating dental intervention and restoration at an accessible intraoral position to be easily inspected or visualized by the patient. According to the World Health Organization recommendations, the examiner recorded a surface as decayed only if it presented with detectably softened or undermined enamel or a softened wall (Bhoopathi et al., 2017). The teeth to be restored should possess healthy periodontium support and clinically show absence of any signs of fistula or current abscess formation.

**Figure 1:**
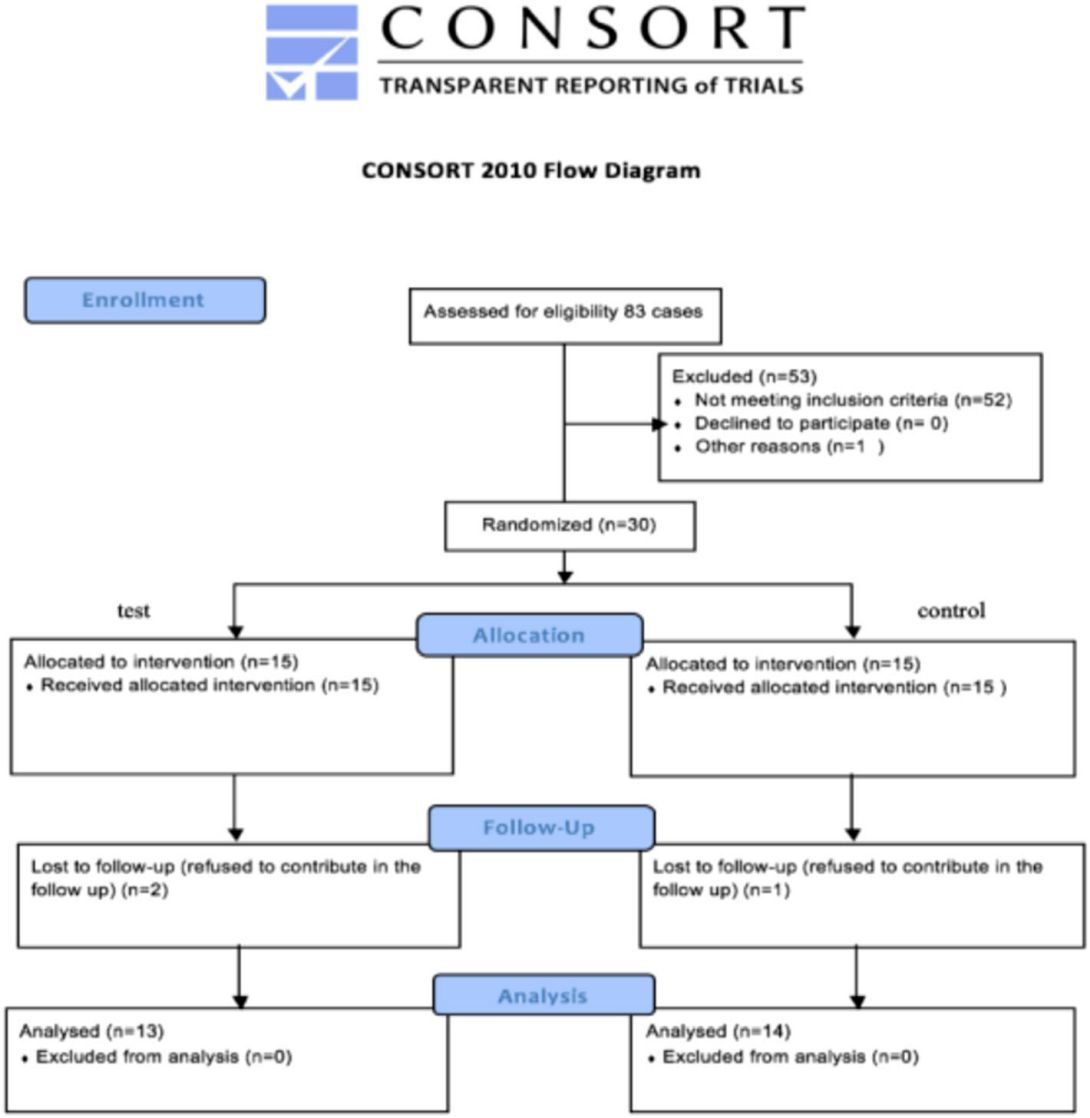
CONSORT diagram depicting participants's enrollment in the study

Eighty‐three patients were reviewed for inclusion in the study, 53 patients were excluded for not meeting the inclusion criteria with final convenience sample composed of 30 participant children as in Figure 1.

All participants were requested to complete an administered questionnaire to establish the sample demographic data. All included patients and their accompanied legal guardian had received a thorough explanation of the experimental rationale, clinical procedure, and any possible complications. Each participant was managed by the same operator throughout the first and the follow‐up visit where the participants were randomly assigned to either investigator using sealed envelopes.

A standard dental procedure was followed with all participants to guard against confounding factors that may affect the study outcome. The cavity was prepared, and the caries lesion was thoroughly excavated following the principle of the conventional adhesive restoration using spoon excavator and #330 pear shaped carbide burs (Midwest®) rotating in a high‐speed handpiece.

Each prepared cavity was carefully inspected to assure absence of any remaining caries then thoroughly cleaned using sterile cotton moistened with normal saline. A self‐etch adhesive (Optibond™ all in one, Kerr, Italy) was used according to the manufacturer instructions in both groups prior final restoration insertion.

Based on the type of the restorative materials used, participants were allocated to Group A, to receive multicolored compomer restoration, or Group B, receiving conventional compomer restoration. Both restorative materials were inserted in increments of 2 mm or less. Each increment was polymerized for 40 s using light curing device. The occlusion was checked using an articulating paper and adjusted accordingly. The restorations were finished using finishing burs (STRAUSS &CO.) and Soflex disks (3M, USA).

### 2.5 Assessment tools

For each participant, the following were assessed before, during, and at the end of the dental procedure as well as at the follow‐up visit:

1. The participant behavior was assessed using the Frankl's behavior rating scale‐FRS (Frankl, 1962). It divides the behavior into four categories according to the Guideline on Behavior Guidance for the Pediatric Dental Patient ranging from definitely negative to definitely positive on the basis of children actions at the dental office.
2. The dental anxiety level using the facial image scale (FIS) consists of a row of five gray scale faces ranging from very happy to very unhappy, and each image has a score from 1 to 5 where 1 represents the most positive response and 5 represents the most negative response. Each child was asked to choose the face that represent his condition at that instant (Alwin, Murray, & Britton, 1991).
3. The dental caries experience was assessed via calculating dmf‐t and DMF‐T indices for primary and permanent teeth, respectively. The tooth scores d/D when decayed, m/M if extracted due to caries, or f/F when had a definitive restoration.
4. The extent of dental plaque accumulation using plaque index (PI) and the gingival index (GI) was used to evaluate the gingival condition. Plaque accumulation was scored on a scale from P0 to P3, where P0 indicates no plaque whereas P3 reflects plaque covering more than one half of the clinical crown. Assessment of the gingival condition was on scale from 0 to 3, where 0 score in case of absence of inflammation whereas 3 score for sever inflammation and spontaneous bleeding (Lőe, 1967).
  Both PI and GI were assessed before the dental procedure at the first visit and at the follow‐up.

All children and their caregiver were given thorough oral hygiene instructions and comprehensive model explanation of horizontal scrub technique for daily oral health care (Duijster, de Jong‐Lenters, Verrips, & van Loveren, 2015; Patil, Patil, & Kashetty, 2014).

A follow‐up recall was scheduled for each participant after 4 weeks to evaluate the inserted restorations and to assess the child oral health status, anxiety level, and degree of cooperation.

### 2.6 Statistical analysis

Data were collected from the patients onto hard copies of data collection forms without showing any nominative information. Subjects were identified by serial study code and initials. These were linked to patient's name in a separate identification log sheet, which was kept in a safe locked place. Two different individuals (PI and co‐investigator) performed the data entry. After verification, data were transferred to statistical database directly.

The collected data were tabulated and statistically analyzed using SPSS version 23 program for Apple Macintosh (Statistical Package for the Social Sciences, SPSS Inc., Chicago, IL, USA).

Chi‐square test was employed to establish gender distribution among groups. Mann–Whitney *U* test was used to test for significance regarding subject's age. Comparison between tests groups in relation to FRS and FIS was done using Mann–Whitney *U* test whereas chi‐square test was used to assess significance within tests groups. Regression analysis was used to test for association between dmf‐t/DMF‐T and FRS/FIS. Plaque index and gingival index mean scores at different treatment stages were tested by Wilcoxon signed‐rank test (within groups), and score difference was calculated to compare between groups and tested using Mann–Whitney *U* test. Spearman's rank was used to test for correlation between Frankl's behavior rating scale at the end of the first visit and the plaque index at the follow‐up visit. Level of significance was tested at *p* ≤ .05.

## 3 RESULTS

From the initial sample consisting of 30 participants, only 27 children (17 male and 10 female) came for the follow‐up visit. Table 2 presents the age mean values, gender distribution, and the dmf‐t/DMF‐T scores of the test groups. Table 3 presents FRS at different stages of the dental treatment of the test groups. The control Group B, which was of older mean age value, showed statistically significant positive FRS before the start of the dental treatment as well as at the follow‐up visit (*p* < .05). Group A subjects expressed a statistically significant negative behavior before the starting of the treatment compared with Group B, which improved significantly to positive and definitely positive behavior according to FRS after the completion of the treatment as well as at the follow‐up visit. FRS assessment at the follow‐up visit reveled positive behavior with statistically insignificant difference between the test groups.

**Table 2:** The age means vales and gender distribution of the test groups

Variable	Group A (n = 13)	Group B (n = 14)	P value
Age	6.38 ± 1.66	7.92 ± 1.49	0.013*
Gender			
Male	9 (69.23%)	8 (57.14%)	0.17
Female	4 (30.76%)	6 (42.85%)	
dmf‐t	7.13 ± 2.83	7.64 ± 3.17	0.65
DMF‐T	4.5 ± 3.69	5.43 ± 4.04	0.715

* Significant.

**Table 3:** Frankl rating score FRS at different stages of the treatment among the test groups

Variable		Group A (n = 13)					Group B (n = 14)				
FRS Time		−−	−	+	++	*P* value	−−	−	+	++	*P* value
V1	Start	0%	61.5%	30.8%	7.7%	0.58	0%	14.3%	78.6%	7.1%	0.002*
	During	0%	23.1%	61.5%	15.4%	0.09	0%	14.3%	78.6%	7.1%	0.002*
	End	0%	7.7%	46.2%	46.2%	0.014*	0%	0%	57.1%	42.9%	0.59
V2	Follow‐up	17.7%	8.3%	66.7%	8.3%	0.01*	14.3%	7.1%	64.3%	14.3%	0.008*

Abbreviations: −−, defiantly negative; −, negative; +, positive; ++, defiantly positive; V1, treatment visit; V2, follow‐up visit.

* Significant.

**Figure 2:**
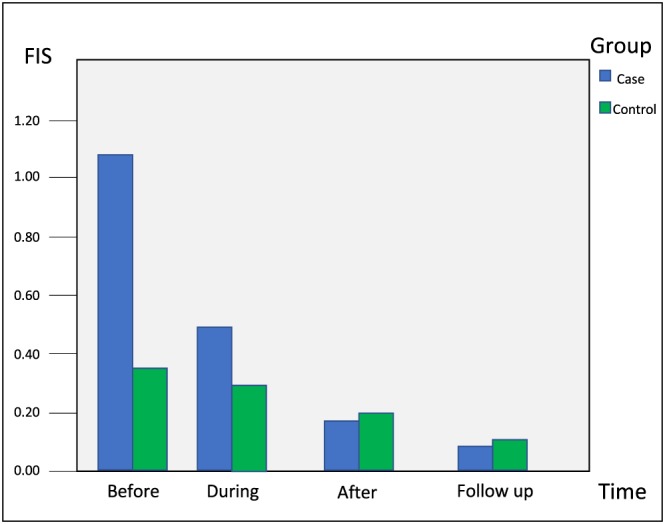
Bar diagram depicting facial image scale at different stages of treatment among the test groups

FIS assessment revealed statistically insignificant difference between the two groups at different treatment stages as depicted in Figure 2.

There were no statistically significant associations between dmf‐t and FRS/FIS at *p* = .59 and *p* = .21, respectively. Similarly, no significant association found between DMF‐T and FRS/FIS at *p* = .89 and *p* = .1, respectively.

Table 4 presents the median and mean values of the plaque index score and gingival index score before the start of the dental procedure at the first visit and the beginning of the follow‐up visit between the test groups. A statistically significant reduction in PI and GI was observed within each group by reaching the follow‐up visit (*p* < .05). Comparing PI mean value at the follow‐up visit revealed a tendency for statistical significance (*p* = .052) as in Table 4.

**Table 4:** Plaque index score and gingival index score comparison at the first visit and the follow‐up visit between the test groups

Variable		Group A (n = 13)		Group B (n = 14)	
		V1	V2	V1	V2
Plaque index	Mean ± *SD*	1.40 ± 0.67^a^	0.57 ± 0.41^b^	1.13 ± 0.33^a^	0.83 **±** 0.38^b^
	Median	1.50	0.50	1.20	0.90
	Score difference	−.50		−.25	
Gingival index	Mean ± *SD*	1.07 ± 0.39^a^	0.57 ± 0.43^b^	1.11 ± 0.22^a^	0.86 **±** 0.42^b^
	Median	1.25	0.75	1.12	0.87
	Score difference	−0.50		−0.37	

*Note*. Different small letter superscripts indicate significance within the row.

Abbreviations: V1, treatment visit; V2, follow‐up visit.

**Figure 3:**
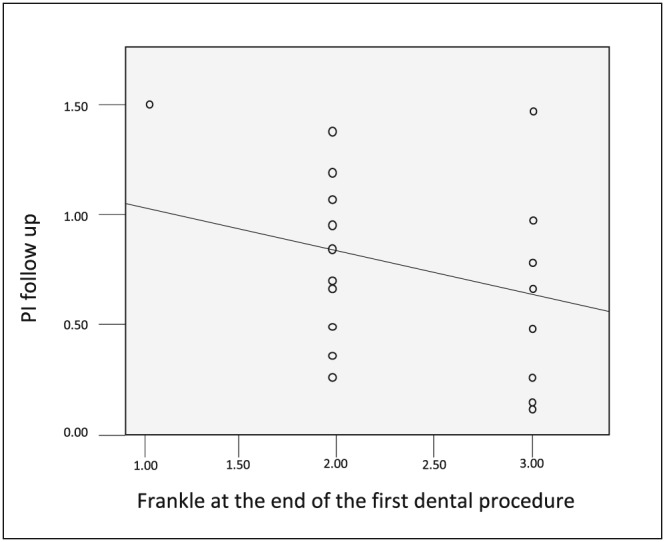
Scatter diagram depicting the correlation between the score of Frankl scale after the treatment at the first visit and plaque index mean score at the beginning of the follow‐up

A strong negative correlation was found between Frankl rating after completion of treatment and plaque index scores in Group A, which found to be statistically significant (*p* = .03) as depicted in Figure 3.

## 4 DISCUSSION

Dental anxiety was found to be common among pediatric dental patients (Klingberg & Broberg, 2007). Multicolored restorations is a dental material, which claimed to have a positive effect on the child behavior (Arora et al., 2014). Thus, the aim of this study was to assess the motivational effect of multicolored restoration on child behavior, anxiety level, and oral hygiene practice.

The initial sample consisted a total of 30 participants, and 10% of them were lost during the follow‐up period, which considered to locate within the acceptable range of attrition in randomized controlled trial's and cohort study (Fewtrell et al., 2008).

Different techniques have been used to measure dental anxiety in children. Some relayed on measuring physiologic signs (as pulse rate), using psychometric scales, assessing the child behavior during the dental visit (as FRS) or using projective method (as FIS). Children behavior was assessed using Frankl's behavior rating scale, which poses up to 93.4% sensitivity and has acceptable validity reaching up to 77.8% (Asokan, Surendran, Punugoti, Nuvvula, & Priya, 2014).

Children self‐rated scales were found to be more precise than those rated by their parents (Paglia et al., 2017). The age of the participants in this study was located in the range of 5–8 years, which known as the preoperational phase. This phase is characterized by development of attention and cognitive abilities, which prepare children for proper social and interpersonal communication. Accordingly, they became more able to express themselves and more prone to be affected by motivational approaches (Radhakrishna et al., 2019). The anxiety level has been frequently assessed using the FIS, which was known for its high reproducibility, simplicity, and validity for both clinical and scientific purposes (Buchanan & Niven, 2002). Studies have shown that FIS is a useful tool to assess dental anxiety even in very young children (Howard & Freeman, 2007; Radhakrishna et al., 2019). The gray scale version was used in this study to avoid any color bias that could accompany colored facial scale index (Buchanan & Niven, 2002).

Dental anxiety has been investigated as a risk factor for dental caries experience (Oba, Dülgergil, & Sönmez, 2009). However, the present study revealed no association between the extent of dental caries and dental anxiety level. This finding is in accordance with a former study, which reported that the severity of dental caries did not result in a significant impact on dental anxiety particularly in children (Taani, El‐Qaderi, & Abu Alhaija, 2005).

The age of the participants was frequently correlated with the dental anxiety level. Several studies reported that dental anxiety became of less intensity with increased age (Oba et al., 2009; Radhakrishna et al., 2019). Similarly, the results of this study conveyed that older children presented more positive attitude toward dental visits. This could be attributed to the evolution of their cognitive ability, enhanced understanding of the surrounding environment with better comprehension of unpleasant situations, and improved ability to deal with anxious conditions (Blomqvist et al., 2013).

The results of the present study cast a new light on younger children showing significantly more tendency to choice multicolored restoration over the conventional tooth‐colored restoration; this finding suggested a useful approach in managing the young children who are the most difficult age group to manage in the dental clinic (Güngör et al., 2016).

The positive improvement in children behavior not only aided to alleviate patients' anxiety but also encouraged to preserve a good condition of the restored teeth (Cianetti et al., 2017). Children sharing in the selection of the restoration color showed better acceptance to the rest of the dental intervention particularly in younger age children (Fishman, Guelmann, & Bimstein, 2006).

The results of the present study are in accordance with the findings of Juliet and Gurunathan (2017), who reported that multicolored restorations could be considered as effective motivational tool where children sight their dental appointment as interacting experience with gleam of different colors in their restorations (Juliet and Gurunathan).

The tools used for assessment of the gingival condition and the oral hygiene were modified Silness and Loe plaque index and gingival index, which were found to be highly sensitive and reproducible (Spolsky & Gornbein, 1996).

The assessment of plaque index yielded a significant reduction per group and a clear tendency toward significance upon comparing between the two groups; this might be due to raised awareness of the children regarding restorations they participated in choosing. It could also be due to the dentist's explanation to the children that the restoration will continue to look good as long as they maintain good oral hygiene measures (Arora et al., 2014). Biesbrock, Walters, and Bartizek (2004) reported that 1‐month reassessment was successful to provide a significant improvement in PI score in 6‐ to 15‐year‐old children reflecting. However, stretching the re‐examination period would ascertain the long‐term finding (Biesbrock et al.).

Even small improvement in plaque control could significantly improve the gingival condition (Esfahanlzadeh, 2011).

In the current study, the gingival index records did not reveal a significant improvement between the two groups. This finding is in accordance with the finding of Esfahanlzadeh, 2011, who reported statistically insignificant change in gingival index score in 6‐year‐old children by the end of the follow‐up period in a dental health education program. This could be attributed to that gingivitis found to be more prominent in older age group during the adolescence stage from 13‐ to 17‐year‐olds owing to the hormonal disturbance, which induces intensive gingival response to the dental plaque and requires extended time period to heal following nonsurgical periodontal therapy (Nakre & Harikiran, 2013). Thus, the effect of hormonal disturbance associated gingivitis is not applied to the present study as the included participants were of younger age group (from 5 to 8‐year‐olds). For both PI and GI, an extended follow‐up period could properly reflect the actual improvement; thus, we recommend stretching the reassessment beyond 4‐week period.

A strong negative correlation was found between Frankl rating scale and plaque index score at the follow‐up visit in Group A; this finding suggested that with improving the children attitude during the dental appointment, a direct enhancement of oral hygiene practice could occur. This finding supports the recommendation of Nguyen, Nguyen, Nguyen, Saag, and Olak (2018) that dentists should expect alleviation of dental anxiety with improved oral health care in children when providing satisfactory dental experience (Nguyen et al.).

We recommend that clinician should try new approaches to manage their pediatric patients, and multicolored dental restoration might be a good solution to reduce patient's anxiety and improve their attitude for a successful treatment outcome and long‐term maintenance.

Further studies using larger sample sizes and extended follow‐up period are needed to affirm significance of the motivational impact of multicolored restorations.

## 5 CONCLUSIONS

Under the limitation of the present study, the following can be concluded:

1. The use of multicolored restorative materials could provide a potential advantage to improve the oral health status and dental care of children.
2. Multicolored restoration might aid in the enhancement of child behavior at the dental clinic particularly in younger age groups.

## CONFLICT OF INTEREST

The authors received no financial support and declared no potential conflicts of interest with respect to authorship and/or publication of this article.

## AUTHOR CONTRIBUTIONS

Melebari L.A. conceived the ideas; Melebari L.A. and Attas S.E. collected the data; Attas S.E. and Arafa A. analyzed the data; and Arafa A. led the writing.
